# Functional dissection of N-terminal nuclear trafficking signals of SETDB1

**DOI:** 10.3389/fcell.2022.1069765

**Published:** 2022-12-20

**Authors:** Jaemin Eom, Kyuheum Jeon, Jung Sun Park, Yong-Kook Kang

**Affiliations:** ^1^ Development and Differentiation Research Center, Korea Research Institute of Bioscience Biotechnology (KRIBB), Daejeon, Korea; ^2^ Department of Functional Genomics, Korea University of Science and Technology (UST), Daejeon, South Korea

**Keywords:** NES, NLS, nuclear import, nuclear export, ATF7IP, PML

## Abstract

SETDB1 is a histone H3-lysine 9-specific methyltransferase that fulfills epigenetic functions inside the nucleus; however, when overexpressed, SETDB1 majorily localizes in the cytoplasm. SETDB1 has a single nuclear-localization-signal (NLS) motif and two successive nuclear-export-signal (NES1 and NES2) motifs in the N-terminus, suggesting that SETDB1 localization is the consequence of a balance between the two antithetic motifs. Here, we performed a series of motif deletions to characterize their effects on the cellular movement of SETDB1. Given the cytoplasmic localization of GFP-SETDB1 in the whole form, without the NES motifs, GFP-SETDB1 was not nuclear, and 3xNLS addition plus NES removal held the majority of GFP-SETDB1 within the nucleus. The results indicated that the cytoplasmic localization of GFP-SETDB1 is the combined result of weak NLS and robust NESs. In ATF7IP-overexpressing cells, GFP-SETDB1 entered the nucleus only in the presence of the NES1 motif; neither the NES2 nor NLS motif was necessary. Since subcellular fractionation results showed that ATF7IP was nuclear-only, an intermediary protein may interact specifically with the NES1 motif after stimulation by ATF7IP. When GFP-SETDB1 had either NES1 or NES2, it was precipitated (in immunoprecipitation) and colocalized (in immunofluorescence) with ATF7IP, indicating that GFP-SETDB1 interacts with ATF7IP through the NES motifs in the nucleus. The regulated nuclear entry of SETDB1 is assumed to set a tight restriction on its abundance within the nucleus, thereby ensuring balanced nuclear SETDB1 levels.

## Introduction

SETDB1 is a histone methyltransferase specific to histone H3 lysine 9 (H3K9). Various transcriptional repressors have been associated with SETDB1 (for review, see (Kang, 2018)). These proteins guide SETDB1 to target genomic loci, rendering the corresponding regions transcriptionally inert through H3K9 methylation and subsequent HP1 recruitment (Schultz et al., 2002). Naturally, SETDB1 fulfills its epigenetic function inside the nucleus. For instance, SETDB1 silences various genomic retroelements, including endogenous viruses (ERVs), thereby contributing to genome stability (Matsui et al., 2010; Liu et al., 2014); SETDB1 epigenetically regulates the structure of the megabase-scale chromatin domain (Jiang et al., 2017); the loss of SETDB1 leads to broad changes in the overall architecture and mechanical properties of the nucleus through genome-wide redistribution of heterochromatin (Zakharova et al., 2022); alternative lengthening of telomeres (ALT) starts from SETDB1-seeded heterochromatin formation, and a loss of SETDB1 abrogates ALT (Gauchier et al., 2019); SETDB1 is involved in maintaining gene silencing on the inactive mouse X chromosome (Minkovsky et al., 2014); and finally, SETDB1 fulfills small RNA-initiated transcriptional gene silencing of gene promoters by inducing local heterochromatin (Cho et al., 2014). Considering the significances of these SETDB1-mediated nuclear processes, there are concerns that if there is no supervising mechanism for nuclear SETDB1 activity, the various epigenetic tasks of nuclear SETDB1 could be impaired by excessive or scant amounts of SETDB1, resulting in a variety of catastrophic consequences.

However, SETDB1 is not restricted to the nucleus and, when overexpressed, is mainly cytoplasmic (Cho et al., 2013; Tachibana et al., 2015). This cytoplasmic retention of overexpressed SETDB1 is considered an additional layer of regulatory mechanisms for SETDB1 function (Cho et al., 2013). In addition, cytoplasmic localization is not solely for exogenous SETDB1 but also for endogenous SETDB1 (Towbin et al., 2012; Cho et al., 2013; Rivera et al., 2015; Tsusaka et al., 2019). For the chromatin modifiers, it is pivotal to securely control their expression and localization, especially when excessive activity can alter chromatin structure and lead to aberrant global gene expression.

The N-terminal region of SETDB1 is known to be associated with the cytoplasmic localization of SETDB1 (Cho et al., 2013; Tsusaka et al., 2019). Anatomizing the protein structure, the N-terminal part of SETDB1 contains functionally opposite motifs—two sequential nuclear export signals (NESs) and a nuclear localization signal (NLS) (Kang, 2015)—signifying the role of SETDB1 as a nucleocytoplasmic shuttle protein. Given that SETDB1 has both NES and NLS motifs at the N-terminus, the relative strength and balance between these motifs may determine the localization of SETDB1, either in the nucleus or cytoplasm. Unfortunately, however, the roles of respective trafficking signals and their combined effect on SETDB1 transport have not been studied until now. In this study, we serially deleted the respective N-terminal motifs to investigate how each affected the nucleocytoplasmic movement of SETDB1. Since ATF7IP (also known as MCAF1 or AM) is a well-known SETDB1 partner and regulator (Wang et al., 2003; Minkovsky et al., 2014; Timms et al., 2016; Tsusaka et al., 2019), we examined whether and which of the N-terminal motifs of SETDB1 specifically responded to the ectopic presence of ATF7IP. Elucidation of the regulatory mechanism of SETDB1 translocation is important for superintending its activity, as it is causally related to a variety of cancers as an oncogene (Lazaro-Camp et al., 2021) and is thus considered a promising therapeutic target for cancer immunotherapies (Griffin et al., 2021).

## Results

### Cytoplasmic localization of SETDB1 is the combined effect of weak NLS and strong NES motifs

**FIGURE 1:**
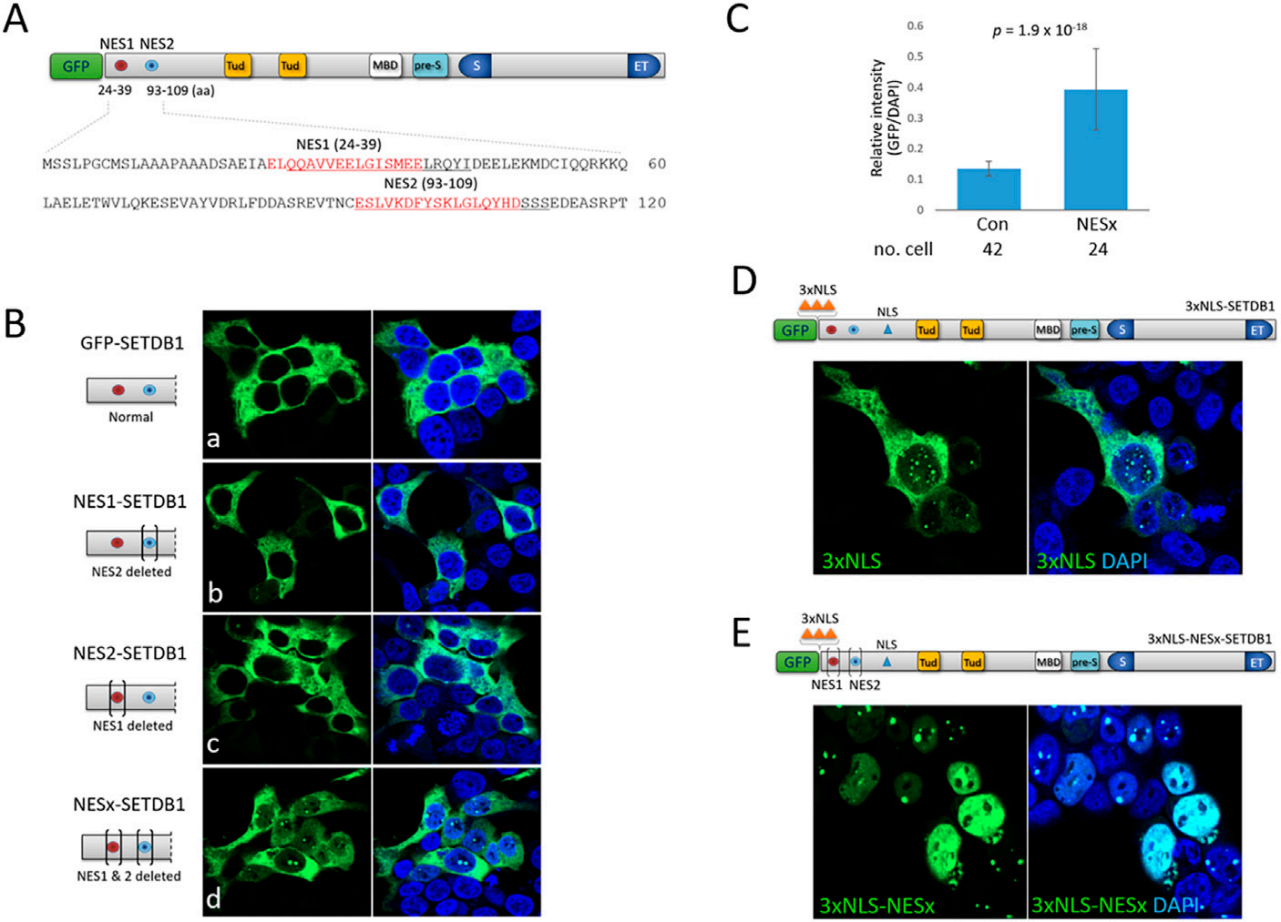
Effect of N-terminal nuclear export and localization signals on subcellular localization of SETDB1 **(A)** Schematic of the SETDB1 protein structure showing two nuclear export signal motifs, NES1 and NES2, in the N-terminus. Individual NES1 and NES2 amino-acid sequences (red) in the N-terminus were deleted (underlined) using CRISPR/Cas9 technology to generate NES1- (NES2 deleted), NES2- (NES1 deleted), and NESx-SETDB1 (both deleted) constructs. Tud, Tudor domain; MBD, methyl-CpG-binding domain; pre-S, pre-SET domain; S and ET, bifurcated SET domain. **(B)** Overexpression of GFP-SETDB1 variants in 293T cells. Brackets in the schematic denote deletions in the indicated NES domains. **(C)** Relative signal intensity of nuclear GFP to DAPI in either GFP-SETDB1 (control) or NESx-SETDB1-expressing 293T cells. The intensity was measured using the histogram tool in Zen software (blue edition v3.4) from Carl Zeiss Microscopy GMBH. The number of cells examined is indicated below. Statistical differences (two-sample *t*-tests) are shown. **(D)** Effect of three copies of NLS (3xNLS, orange-colored triangles) in the N-terminus on subcellular localization of SETDB1. The resulting 3xNLS-SETDB1 construct was expressed in 293T cells. **(E)** The movement of the NES-deleted (NESx) 3xNLS-SETDB1 construct (3xNLS-NESx-SETDB1) in 293T cells. Images were obtained using a confocal microscopy (Carl Zeiss LSM800).

To identify factors that are implicated in the nucleocytoplasmic trafficking of SETDB1, we examined the efficacy and strength of the two separate NES motifs, NES1 and NES2, in the N-terminus of SETDB1 (Figure 1A). We removed each or both NESs from GFP-SETDB1 to generate NES1- (NES2 deleted), NES2- (NES1 deleted), and NESx-SETDB1 (both deleted) constructs. All SETDB1 variants we constructed were GFP-tagged at the N-terminus and, hereafter, we omitted the ‘GFP’ in the vector names for convenience, except for the control GFP-SETDB1. Overall, the expression levels of SETDB1 variants ranged from four-to ten-fold of endogenous SETDB1 (Supplementary Figure.S1). When expressed in 293T cells, agreeing with previous result (Cho et al., 2013), GFP-SETDB1 was mainly cytoplasmic. Neither NES1- nor NES2-SETDB1 expression patterns, nor their density, were noticeably different from those of normal GFP-SETDB1 (Fig. 1Bb and Bc). In NESx-SETDB1 cells, solid nuclear dots, which are rare in GFP-SETDB1-expressing cells, were detected more frequently (71% ± 0.13, *n* = 261 cells; Fig. 1Bd). Furthermore, while the NESx-SETDB1 signal was mostly cytoplasmic, it was also shown to be dimly diffuse in the nucleus. The GFP intensity per unit area of NESx-SETDB1-expressing cells was significantly higher than that of GFP-SETDB1 cells (0.393 ± 0.133 vs. 0.135 ± 0.024, *p* = 1.873 × 10^–18^; Figure 1C). These results indicate that SETDB1 accumulates in the nucleus only when both NES motifs are absent, suggesting that the respective NES motifs operate to send SETDB1 back to the cytoplasm once it enters the nucleus.

SETDB1 is predominantly cytoplasmic even in the absence of NES motifs, which indicates that the nuclear import of SETDB1 is strictly checked and that the less efficient NLS of SETDB1 may play a part. To boost the nuclear import of SETDB1, we added three copies of NLSs (3xNLS) from the simian virus 40 large T-antigen (Kalderon et al., 1984) to the N-terminus of SETDB1, yielding a 3xNLS-SETDB1 construct. Although a part of the 3xNLS-SETDB1 signal was still cytoplasmic in 293T cells, the motif succeeded in driving a large quantity of SETDB1 into the nucleus, displaying many dots of variable size (Figure 1D). In addition, we tested whether cytoplasmic 3xNLS-SETDB1 was a subset that had gained access to the nucleus but was expelled by NESs. When the NES motifs were deleted from 3xNLS-SETDB1, the resulting 3xNLS-NESx-SETDB1 was totally nuclear, displaying multiple dots along with a diffuse nucleoplasm signal and only a faint cytoplasmic signal, if any (Figure 1E). Together, these results indicate that SETDB1 remaining in the cytoplasm is the combined result of the inefficacious NLS (see below) and relatively robust NESs.

### Nuclear ATF7IP encourages cytoplasmic SETDB1 to enter the nucleus indirectly

**FIGURE 2:**
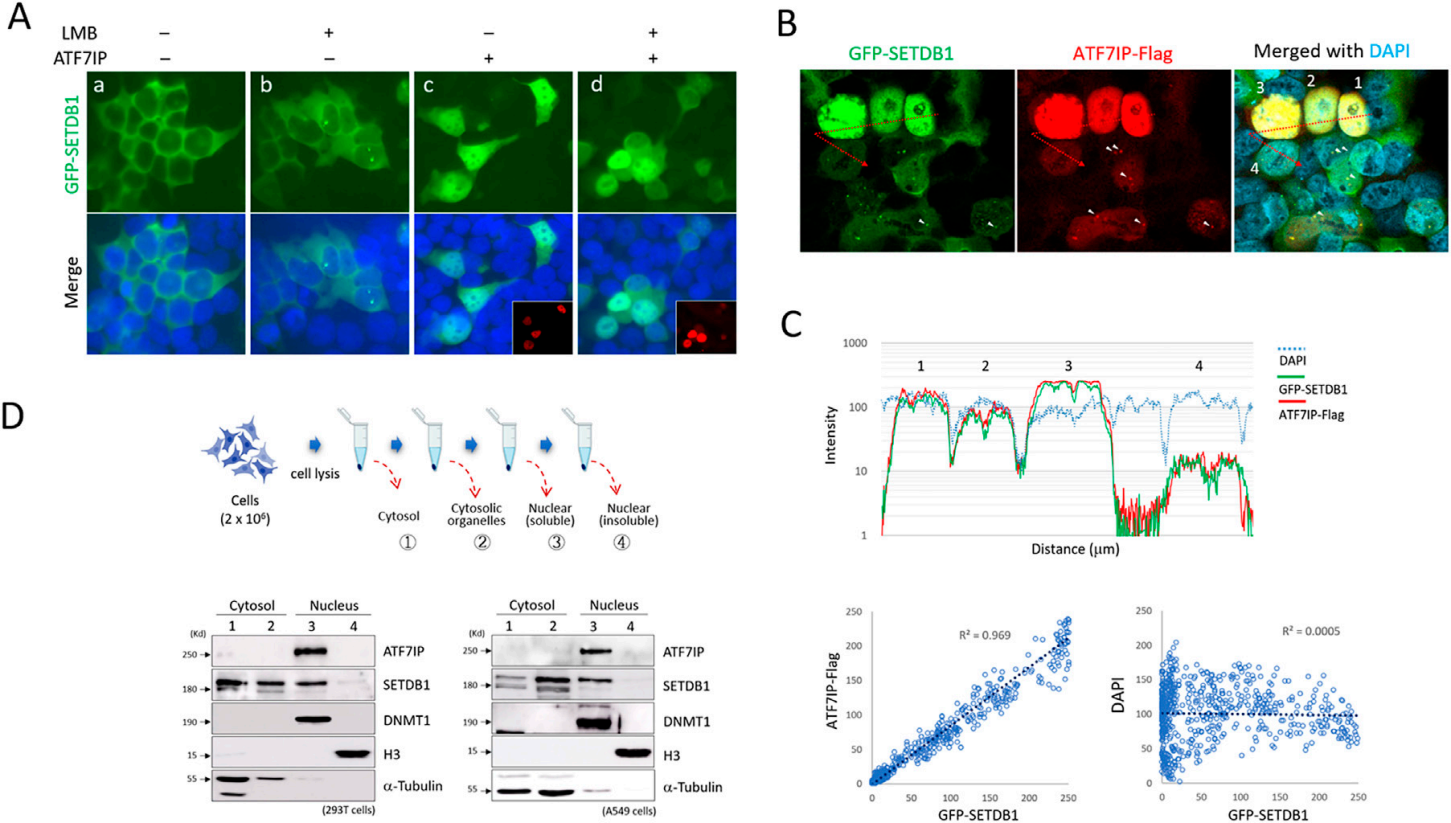
Induction of cytoplasmic GFP-SETDB1 into the nucleus by ATF7IP **(A)** Nuclear localization of GFP-SETDB1 after ATF7IP-Flag overexpression. ATF7IP overexpression stimulates GFP-SETDB1 to enter the nucleus, regardless of leptomycin B (LMB) treatment. The insets in **(c** and **d)** indicate ATF7IP-Flag IF images. **(B–C)** Correlation between ATF7IP-Flag and nuclear GFP-SETDB1 expression levels. In **(C)**, a signal intensity profile (top) and scatter plots (bottom) were produced from the merged picture in **(B)** using ‘Profile” tool offered in the Zen program (v3.4) from Carl Zeiss microscopy. It reveals that the density of nuclear GFP-SETDB1 signal closely correlates with that of ATF7IP-Flag signal. Scatter plots were created using fluorescence intensity values per micrometer of distance. The cells in **(B** and **C)** are identically numbered for analysis (1–4). The colocalization of the dotted GFP-SETDB1 and ATF7IP-Flag signals is shown in **(B)** by the arrowheads. **(D)** Western blotting results after subcellular fractionation in 293T (bottom, left) and A549 cells (bottom, right). Fraction procedure is illustrated (top) in the order of cytosolic (1), cytosolic organelle (2), nucleoplasmic (3), and insoluble chromatin fractions (4). DNMT1, a nuclear marker; H3, an insoluble chromatin marker; α-tubulin, cytosolic marker.

When overexpressed, ATF7IP alters the cellular localization of SETDB1 (Timms et al., 2016; Tsusaka et al., 2019). When GFP-SETDB1 and ATF7IP-Flag were expressed separately in 293T cells, ATF7IP-Flag was clearly nuclear, whereas GFP-SETDB1 was cytoplasmic. In the presence of leptomycin B (LMB), a CRM1/XPO1 inhibitor that blocks protein transport to the cytoplasm (Kudo et al., 1999), the GFP-SETDB1 signal mostly remained in the cytoplasm, except for frequent dots in the nucleus (43% ± 0.21, *n* = 327 cells; Fig. 2Ab), which was also shown in a previous study (Cho et al., 2013). However, when the ATF7IP-Flag was co-expressed, GFP-SETDB1 gained access to the nucleus regardless of LMB treatment (Fig. 2Ac and 2Ad). Because the nuclear intensity of GFP-SETDB1 increased along with the ATF7IP intensity, it appeared that the signal intensity of nuclear SETDB1 was proportionate to that of ATF7IP-Flag (Figure 2B). The fluorescence intensity profile revealed that the signals for GFP-SETDB1 and ATF7IP-Flag were both synchronized (Figure 2C, top), and that the signals for GFP-SETDB1 and ATF7IP-Flag were strongly correlated (coefficient of determination, R2, = 0.969), but not with the signals for DAPI (R2 = 0.0005; Figure 2C, bottom). Additionally, the ATF7IP signal typically overlapped with the dotted GFP-SETDB1 signal, which was commonly seen in the nucleus when ATF7IP-Flag expression levels were low (Figure 2B).

These results indicate that nuclear ATF7IP is implicated in the import of cytoplasmic SETDB1, and raised a possibility of ATF7IP shuttling between the nucleus and the cytoplasm. To test this possibility, we examined the cellular localization of endogenous ATF7IP by subcellular fractionation. The results showed that ATF7IP was nuclear only, whereas SETDB1 was both nuclear and cytoplasmic in 293T and A549 cells (Figure 2C) as previously shown in mouse embryonic fibroblasts (Cho et al., 2013). Notably, SETDB1 is majorily detected in the nucleus of mouse embryonic stem cells and NIH3T3 cells, which suggests that cellular localization of SETDB1 is cell type-specific. The immunofluorescence (IF) and cell fractionation results argue against the nucleocytoplasmic commuting of ATF7IP. Since ATF7IP is confined solely in the nucleus, it is unlikely that ATF7IP directly transports SETDB1 into the nucleus. These results indicate that the nuclear import of GFP-SETDB1 depends on the ectopic expression of ATF7IP; however, it is unknown how nuclear ATF7IP encourages cytoplasmic SETDB1 to enter the nucleus.

### The NES1 motif is required for ATF7IP-mediated nuclear localization of SETDB1

**FIGURE 3:**
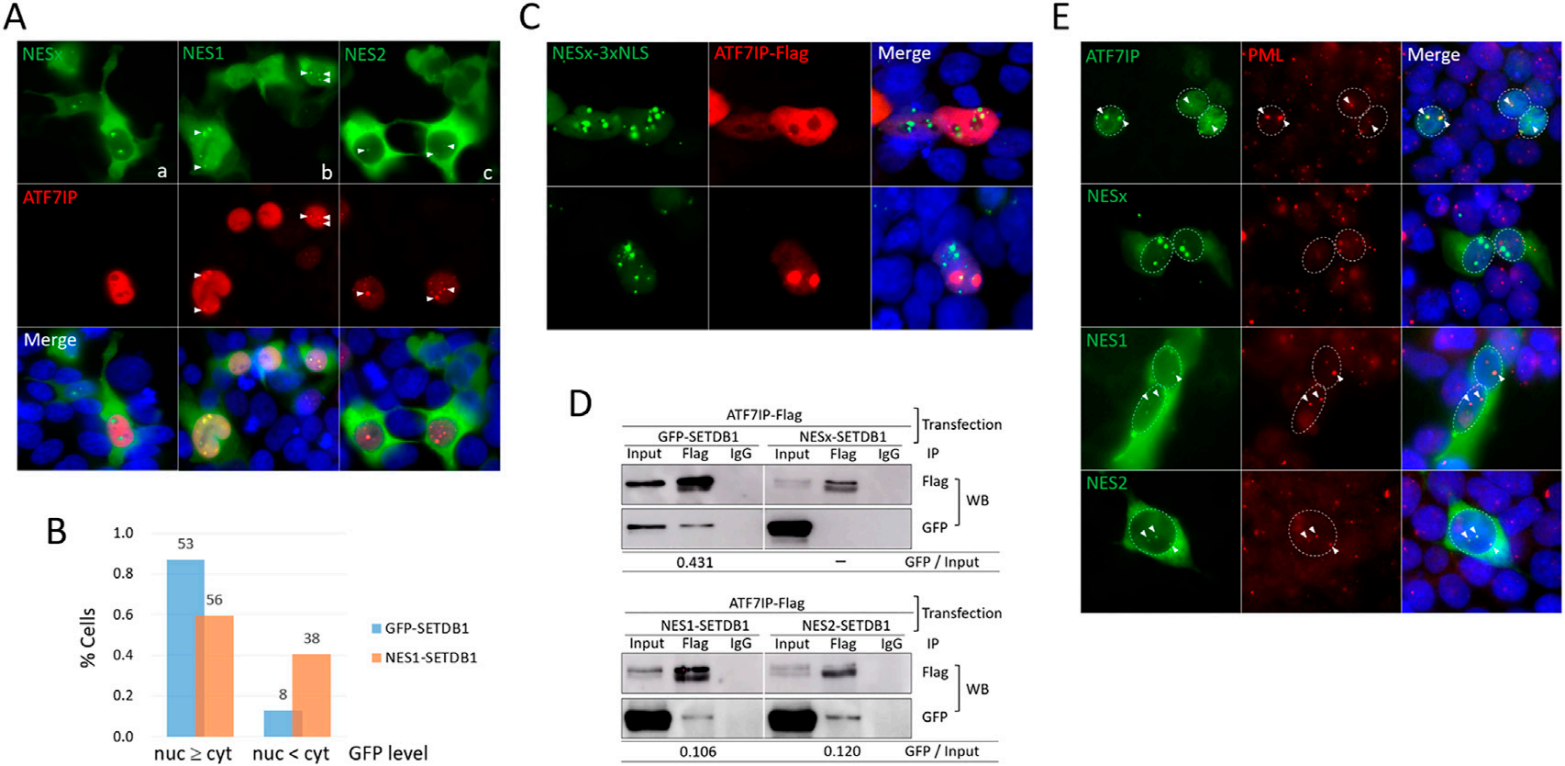
The NES1 motif is necessary for ATF7IP-mediated nuclear localization of SETDB1 **(A)** Effect of NES motifs on SETDB1 movement in ATF7IP-overexpressing 293T cells. Arrowheads point to nuclear dots overlapped between cells expressing NES variants and ATF7IP-Flag. **(B)** Comparison of ATF7IP-induced nuclear localization efficiency between GFP-SETDB1 and NES1-SETDB1. The fractions of cells with high (nuc ≥ cyt) or low (nuc < cyt) nuclear GFP levels compared with cytoplasmic GFP levels were calculated. The counts in the bars indicate the number of cells examined. The results of two independent experiments were summed. **(C)** Discordant localization of NESx-3xNLS-SETDB1 and ATF7IP in the nucleus. **(D)** Immunoprecipitation (IP) of the NES variants using an anti-Flag antibody. 293T cells were co-transfected with ATF7IP and the indicated NES variants prior to IP. The IP efficiency was obtained by calculating the precipitated GFP band density relative to the input GFP band density (GFP/input). **(E)** PML localization of NES variants in 293T cells. Cells were co-transfected with the indicated NES variant constructs and ATF7IP-Flag and stained using an anti-PML antibody. Nuclear GFP dots in NES-variant-expressing cells were assumed to be induced by ATF7IP overexpression. Colocalization of PML and GFP signals is indicated by arrowheads.

ATF7IP binds to the N-terminal (1–109 aa) of SETDB1, where the two NES motifs are present, and interferes with the export of nuclear SETDB1 (Tsusaka et al., 2019). Since NESx-SETDB1 retains two-thirds of the N-terminal (72/109 aa; see Figure 1A) by pinpoint deletions, there is still a chance that ATF7IP binds to the remaining N-terminal part lacking the NES motifs. However, when ATF7IP and NESx-SETDB1 were co-expressed, ATF7IP overexpression did not alter the cellular localization of NESx-SETDB1 (Fig. 3Aa). Next, we examined the respective NES motifs. Interestingly, NES1- and NES2-SETDB1 showed different expression patterns in 293T cells. NES1-SETDB1 exhibited a pattern similar to that of GFP-SETDB1 in that it was diffusely present in the ATF7IP-positive nucleus (Fig. 3Ab); however, the proportion of cells with dense nuclear signals was not as much higher in the NES1-SETDB1 as in the GFP-SETDB1 control (60% vs 87%; Figure 3B). In contrast, NES2-SETDB1 was mostly cytoplasmic, regardless of ATF7IP-Flag expression, and resembled NESx-SETDB1 (Fig. 3Ac). We further inspected NES variants and ATF7IP-Flag-expressing cells after LMB treatment; if these NES variants ride a “first-import-later-export” mechanism, they would accumulate in the export-blocked nucleus. However, LMB treatment did not alter the localization pattern of the NESx variant, suggesting that the import of NESx and NES2 variants is restricted (Supplementary Figure S2). This indicates that without ATF7IP overexpression, it is not easy for the NES variants to enter the nucleus and that for ATF7IP-mediated SETDB1 import, the NES1 motif is required. This suggests that nuclear export is not the only task of the NES motifs; they may also play a role in the ATF7IP-mediated nuclear import of SETDB1.

### NES motif is required for the association of SETDB1 with ATF7IP and PML

All three NES variants, NESx, NES1, and NES2-SETDB1, showed a dotted signal in the ATF7IP-positive nucleus (Figure 3A). The dots of the NES1 and NES2 variants overlapped with ATF7IP-Flag, whereas those of the NESx variant did not. Even when a substantial amount of NESx-SETDB1 was forced into the nucleus by adding 3xNLS, NESx-SETDB1 dots did not overlap with the ATF7IP-Flag signal (Figure 3C). To determine whether this signal overlap indicated their physical association, we performed immunoprecipitation (IP) experiments using an anti-Flag antibody. Consistent with the IF results, ATF7IP precipitated with NES1- and NES2-SETDB1 as well as GFP-SETDB1, but not with NESx-SETDB1 (Figure 3D). Compared with GFP-SETDB1, which possesses both NES motifs, the NES1 and NES2 variants showed weakened binding to ATF7IP (25% and 28% of the GFP-SETDB1 control, respectively). In addition, promyelocytic leukemia protein (PML)-nuclear body (PML-NB) was previously identified as a site where both ATF7IP (Sasai et al., 2013) and SETDB1 (Cho et al., 2011) bind. Indeed, the ATF7IP dot signal corresponded to the PML signal in the 293T cells (Figure 3E). Fluorescence intensity profile analysis showed that dot signals of both NES1 and NES2 variants were also localized in PML-NB, whereas those of the NESx variant were not (Figure 3E and see also Supplementary Figure S3). Together, these results indicate that SETDB1 associates with ATF7IP in PML-NBs *via* at least one of the NES motifs.

### The NLS motif is dispensable for ATF7IP-mediated nuclear entry of SETDB1

**FIGURE 4:**
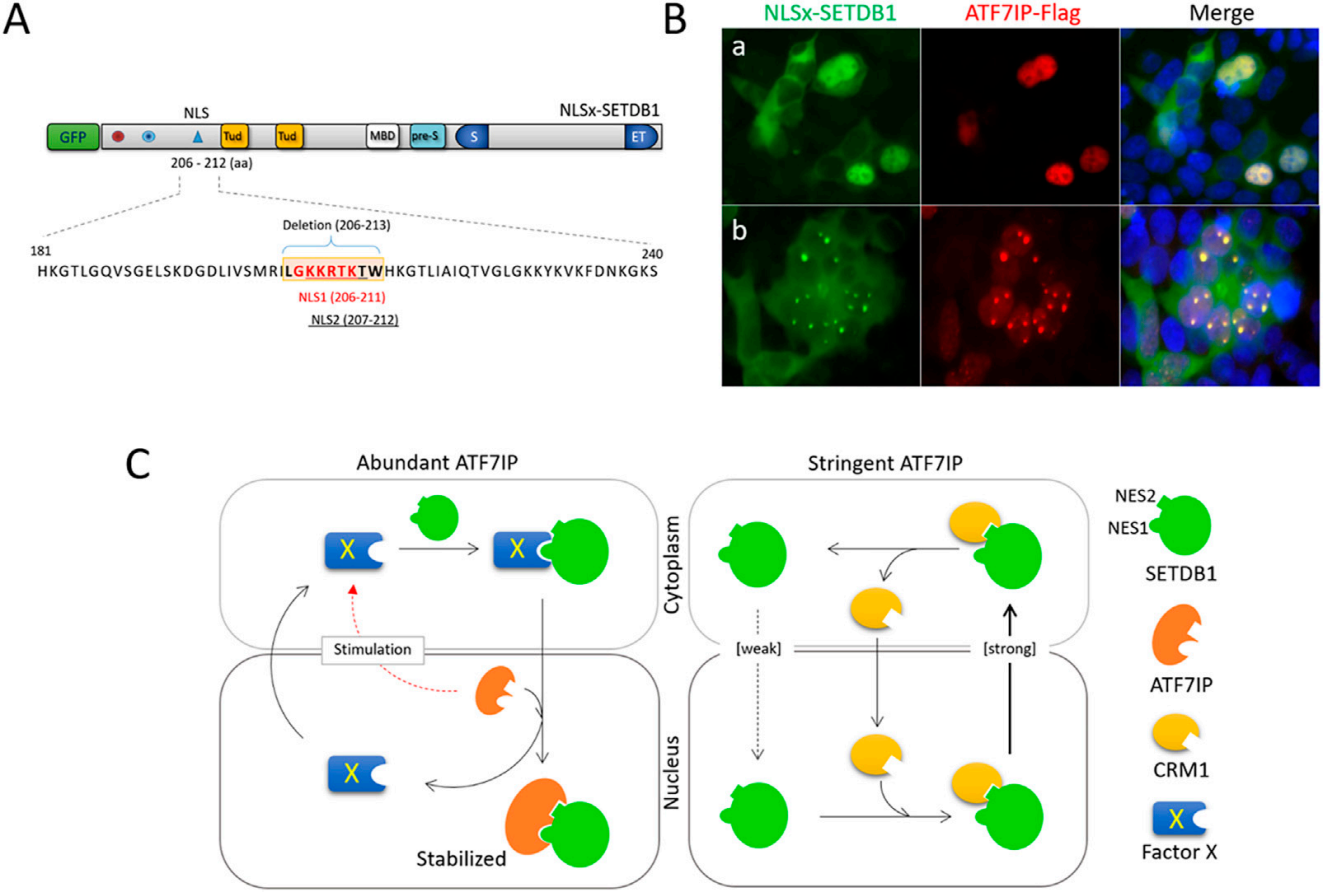
The NLS motif is dispensable for ATF7IP-stimulated movement of SETDB1 **(A)** Deletion of the NLS motif in GFP-SETDB1. Two almost overlapping NLS motifs (NLS1 in red and NLS2 underlined) in the SETDB1 N-terminus were deleted to generate NLSx-SETDB1 using CRISPR/Cas9 technology. **(B)** Effect of the NLS motif on SETDB1 movement in ATF7IP-overexpressing 293T cells. **(C)** A model of SETDB1 movement in an ATF7IP abundant or stringent condition.

Finally, we analyzed the role of the NLS motif in SETDB1 trafficking. Two overlapping NLS motifs with single amino acid differences were present in the SETDB1 N-terminus, which persuaded us to consider it as a single NLS. We deleted the encompassing sequence to generate NLSx-SETDB1 (Figure 4A). As expected, NLSx-SETDB1 was exclusively cytoplasmic in the 293T cells (Supplementary Figure S4). However, when co-expressed with ATF7IP-Flag, NLSx-SETDB1 was primarily localized in the nucleus (Fig. 4Ba), with frequent nuclear dots (Fig. 4Bb). The efficacy of NLSx-SETDB1 for nuclear access was similar to that of normal GFP-SETDB1 (Figure 2B). Since SETDB1 can access the nucleus without the NLS motif, the NLS motif is considered to be dispensable for ATF7IP-involved nuclear import of SETDB1. In summary, we analyzed the role of each of the N-terminal NES1, NES2, and NLS motifs in the nucleocytoplasmic movement of SETDB1 by a separate or combined pin-point deletion of respective motifs. We made new findings from this attempt as following: 1) the NLS motif is relatively weak in performance whereas the NESs are robust, and the cytoplasmic localization of GFP-SETDB1 is the outcome of equilibrium of the performance of weak NLS and robust NESs; 2) The NES1 motif, but not the NES2, is necessary for GFP-SETDB1 to move into the nucleus in the abundant presence of ATF7IP; 3) The NLS motif is not required for ATF7IP-mediated nuclear entry of SETDB1; 4) ATF7IP stays in the nucleus only and does not commute between the nucleus and cytoplasm; and 5) ATF7IP binds to either of the NES1 and NES2 motifs of SETDB1 in the nucleus.

## Discussion

SETDB1 has both NLS and NES motifs together in its N-terminus. It implies that the cellular localization of SETDB1 is the consequence of a balance between these two motifs with opposite effects. The fact that GFP-SETDB1 mostly stays in the cytoplasm reflects that the equilibrium leans towards the NES function—a delayed import against a quick export. Our results from serial deletions of the NES and NLS motifs support this cellular movement of GFP-SETDB1 in 293T cells. The NLS motif operates poorly and is thus unable to shuttle enough SETDB1 into the nucleus, according to the results of LMB treatment (Figure 2A). In contrast, the NES motifs were robust enough to rapidly force barely infiltrated SETDB1 out of the nucleus (Figures 1B,E). Therefore, we conclude that owing to the composite nature of the motifs, SETDB1 alone cannot easily access the nucleus or stabilize once entered. This suggests that whenever SETDB1 accesses the nucleus, it requires a partner protein that bestows an active NLS function on SETDB1 upon binding.

ATF7IP is known to help SETDB1 stay in the nucleus (Timms et al., 2016; Tsusaka et al., 2019). ATF7IP was proposed to promote SETDB1 entry into the nucleus by transporting SETDB1 from the cytoplasm (Tsusaka et al., 2019). We observed that ATF7IP overexpression stimulated the nuclear import of GFP-SETDB1 through an ‘indirect’ pathway in which an intermediary factor may be involved. Although we currently do not know the identity of this factor, the presumption of its presence is logical considering that ATF7IP is retained in the nucleus, playing no commuter to the cytoplasm, as evidenced by our IF results and others (Ichimura et al., 2005; Sasai et al., 2013; Tsusaka et al., 2019) as well as our cell fractionation results (Figure 2C). Whatever this yet-to-be-characterized protein may be, it requires the NES1 motif for the binding and delivery of SETDB1 to the nucleus.

In resting 293T cells, the cellular localization of NES1-SETDB1 was not different from that of the NESx- and NES2-SETDB1 variants (Figure 1B). However, upon ATF7IP overexpression, NES1-SETDB1 moved into the nucleus, whereas NESx- and NES2-SETDB1 largely remained in the cytoplasm indifferently (Figure 3A). The binding affinities of NES1 and NES2 for ATF7IP did not differ according to the IP results (Figure 3C). Therefore, the similar binding disposition of NES1 and NES2 to ATF7IP cannot explain their contrasting capabilities for nuclear transport. Similarly, if ATF7IP can directly import cytoplasmic SETDB1 into the nucleus, both the NES1 and NES2 motifs should have shown equivalent performance in SETDB1 relocation. This is another indication of the existence of an intermediary protein that helps SETDB1 to move to the nucleus in the direction of ATF7IP.

Based on our results of dissecting the N-terminal structure of SETDB1, we propose a model for nucleocytoplasmic trafficking of overexpressed SETDB1 in 293T cells (Figure 4C). Under stringent ATF7IP conditions, GFP-SETDB1 proteins are mostly cytoplasmic due to inefficient NLS motifs along with relatively robust NESs. SETDB1 proteins that enter the nucleus are usually quickly removed from the nucleus by exporter CRM1, which recognizes the NES motifs of SETDB1. With abundant ATF7IP, a yet-to-be-characterized factor X is stimulated to bring GFP-SETDB1 to the nucleus. Factor X recognizes the NES1 motif of cytoplasmic GFP-SETDB1 and delivers it to the nucleus. Once GFP-SETDB1 is imported, ATF7IP, which has a higher binding affinity than factor X for NES motifs, immediately replaces factor X to bind SETDB1. ATF7IP outcompetes CRM1 for SETDB1 binding, and the ATF7IP-SETDB1 interaction stabilizes SETDB1 in the nucleus, facilitating SETDB1 to perform its work at target chromatin loci and nuclear organelles like PML-NBs. Alternatively, GFP-SETDB1 free from protein X is attracted to PML-NB where ATF7IP is abundant and is stabilized.

Our findings provide insight into the regulation of subcellular localization of SETDB1 in the context of abundant ATF7IP. As SETDB1 overexpression and its oncogenic role is evident in a broad range of cancers, SETDB1 is spotlighted as a promising target for therapeutic interventions (Karanth et al., 2017; Lazaro-Camp et al., 2021). Therefore, the understanding of the regulatory mechanism of SETDB1 transport and, hopefully, the elucidation of partner protein(s) involved in nuclear entry of SETDB1 would help to design multiple upstream and downstream therapeutic targets of SETDB1 in its oncopathgenic pathway.

## Methods and materials

### Vector construction

For deletions of the NES and NLS sequences in GFP-SETDB1 expression plasmid (Cho et al., 2013), we used CRISPR/Cas9 technology. Thirty mM of sgRNA was preincubated with 30 mM of Cas9 nuclease (Toolgen) at RT for 10 min before reaction with GFP-SETDB1 plasmid at 37°C for additional 15 min. After spin-column purification, Cas9-generated sticky ends of the plasmid were repaired by T4 DNA polymerase (New England Biolabs, NEB) and Klenow enzyme (NEB). The resulting plasmid was then self-ligated using T4 DNA ligase (NEB) and T4 DNA ligase buffer (NEB). For preparing the NESx variant, the NES2-SETDB1 plasmid harboring the NES1 deletion was first constructed and, with this plasmid as template, we repeated the deletion process for the NES2 sequence. Information on the sgRNA sets used is as following: 5′-aga​gau​ugc​uga​gcu​gca​gcagg-3′ and 5′-acu​ucg​uca​gua​cau​uga​ugagg-3′ for NES1 deletion; 5′-agu​gac​uaa​cug​uga​guc​uuugg-3′ and 5′-gua​uca​uga​cag​uag​cuc​ugagg-3′ for NES2 deletion; and 5′-aua​guc​agc​aug​cgg​auu​cuggg-3′ and 5′-agg​acu​aag​aca​ugg​cac​aaagg-3′ for NLS deletion.

### Cell culture, transfection, and leptomycin-B treatment

293T cells were grown in Dulbecco’s modified Eagle’s medium (Gibco) supplemented with 10% fetal bovine serum (FBS), 0.5% non-essential amino acids, 100 units/ml penicillin and 0.1 mg/ml streptomycin at 37°C in a humidified atmosphere of 5% CO_2._ For transfection, Lipofectamine 3000 (Invitrogen) was used according to the manufacturer’s instruction. For Leptomycin-B (LMB) treatment, 293T cells were incubated in 200 nM LMB (LC Laboratories) for 3 h.

### Antibodies and immunostaining

The list of antibodies we used was as follows: anti-Flag (F3165, Sigma), anti-GFP (sc-9996, Santacruz), anti-ATF7IP/MCAF1 (A300-169A, Bethyl), anti-SETDB1 (11231-1-AP, Proteintech), anti-H3 (ab1791, abcam), anti-DNMT1 (custom-made) and anti-α-Tubulin (sc-5286, Santacruz).

For immunostaining, 293T cells were cultured overnight on a poly-l-lysine (0.01%; sigma)-precoated coverslip and, after a brief rinse in PBS, fixed in 4% formaldehyde at RT for 10 min. The cells were washed three times each for 10 min with PBST (PBS supplemented with 0.05% Tween-20) and then permeabilized in PBST containing 0.2% Triton X-100 (MP Biomedicals) for 10 min at RT. The cells were blocked in PBST containing 1% BSA at RT for 1 h before incubation with primary antibodies at 4°C overnight. After washing three times with PBST, the cells were incubated with Alexa Flour 488- or 594 –conjugated secondary antibodies at RT for 1 h. The stained coverslip was mounted on a slide glass with a mounting medium containing 4’, 6-diamidino-2-phenylindole (DAPI, Vectashield). Samples were observed with Carl Zeiss Axiovert 200 _M_ fluorescence microscope equipped with ApoTome. Most staining experiments were repeated at least three times and images were captured digitally using different filter sets and analyzed using Zen software (Car zeiss microscopy, blue edition, v3.4). As the figure exemplary of the IF result, an IF image that was typical (>70% in fraction) of immunostained cells was chosen.

### Immunoprecipitation and western blot analysis

To obtain whole cell lysates, 293T cells were harvested 48 h after co-transfection of ATF7IP-Flag and SETDB1 NES variant, and bursted in a lysis solution (1% NP-40, 50 mM Tris-HCl (pH 8.0), 150 mM sodium chloride, 2 mM EDTA, PMSF, and protease inhibitor cocktail) on a rotator for 1 h at 4°C. The lysates were centrifuged at 13,000 x g for 20 min and the supernatant was collected as a whole cell lysate sample. For IP, the whole cell lysates were incubated in the lysis buffer with anti-Flag (Sigma) or normal mouse IgG (Santacruz) as the negative control at 4°C overnight. Protein G magnetic beads (Bio-rad) were pre-washed three times with the lysis buffer, added to the antibody mixture, and incubated for additional 4 h at 4°C. Beads were washed 3 times each for 10 min and boiled in SDS-Page loading buffer (Biosesang) at 95°C for 10 min. The supernatants and inputs were subjected to the Western blot analysis. For Western blot analysis, 293T cells were harvested and lysed in lysis solution at 4°C for 1 h followed by a centrifugation at 13,000 x g for 20 min. Protein concentrations were measured using Bradford solution (Bio-rad), and the lysates were boiled in SDS-Page loading buffer (Biosesang) at 95°C for 10 min. The denatured samples were electrophoresed on a SDS-PAGE gel and transferred to nitrocellulose membrane (Amersham). After transfer, the membrane was blocked with 5% skim milk in TBST at RT for 1 h and incubated with a primary antibody overnight at 4°C. The membrane was then washed three times with TBST each for 15 min and incubated with appropriate HRP-conjugated secondary antibodies at RT for 1 h. The membrane was washed three times and the signal was detected using a chemiluminescent substrate (Amersham).

### Subcellular fractionation

Cells were harvested and fractionation was performed using Subcellular Protein Fractionation Kit (ThermoFisher) according to the manufacturer’s instruction. In brief, 2 × 10^6^ cells were harvested and washed twice with ice-cold PBS. The cells were then incubated in 200 μl of Cytoplasmic Extraction Buffer (CEB) on a rotator for 10 min at 4°C, and centrifuged at 500 x g for 5 min. The supernatant as cytoplasmic extract was transferred to a pre-chilled tube on ice. The remaining pellet was resuspended in 200 μl of ice-cold Membrane Extraction Buffer (MEM) and vortexed for 5 s. The sample was then incubated for 10 min at 4°C followed by centrifugation at 3,000 x g for 5 min. The supernatant as membrane extract was transferred to a pre-chilled tube on ice. For nuclear extraction, 100 μl of ice-cold Nuclear Extraction buffer (NEB) was added to the sample. After brief vortexing, the sample was incubated for 30 min at 4 °C before centrifugation at 5,000 x g for 5 min. The supernatant as nuclear extract was transferred to a pre-chilled tube on ice. Finally, the remaining pellet was incubated in 100 μl of NEB containing 5 mM CaCl_2_ and 300 units of micrococcal nuclease for 15 min at RT and centrifuged 16,000 x g for 5 min. The supernatant as the chromatin-bound nuclear extract was transferred to a pre-chilled tube on ice. All buffers contain protease inhibitors, and centrifugation was performed at 4°C. For western blot analysis, the concentration of the cytosolic fraction was determined and 50 μg of cytosolic fraction was used in gel loading. The other fractions were loaded in proportion to the volume of 50 μg cytosolic fraction.

## Data Availability

The original contributions presented in the study are included in the article/Supplementary Material, further inquiries can be directed to the corresponding author.

## Abbreviations

NES and NLS, nuclear export and localization signal;LMB, leptomycin B; PML-NB, polymyelocytic leukemia protein-nuclear body.
