# Supplementary material for: Functional dissection of N-terminal nuclear trafficking signals of SETDB1
Source: Front Cell Dev Biol. 2022 Dec 20;10:1069765. doi: 10.3389/fcell.2022.1069765 (PMC9807615; doi:10.3389/fcell.2022.1069765)
Supplement: Supplementary file 1 [file DataSheet1.PDF]

Supplementary Figure S1

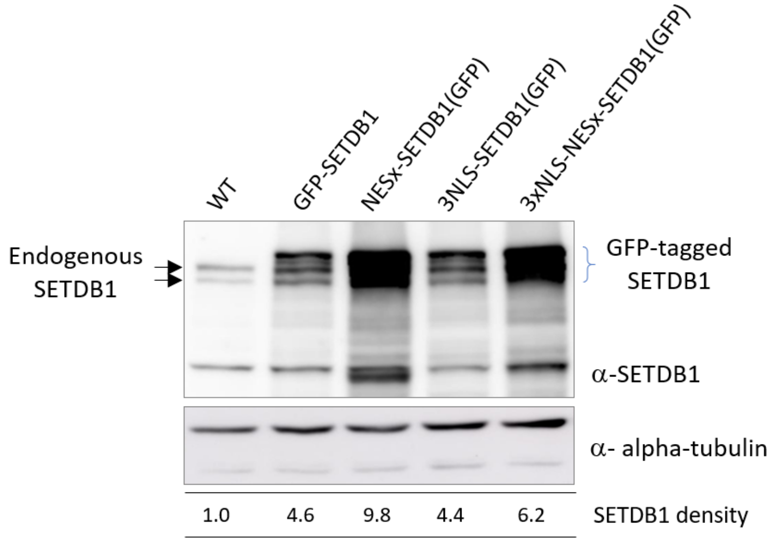

Figure S1. Protein levels of SETDB1 variants relative to the endogenous SETDB1 (arrows).

Supplementary Figure S2

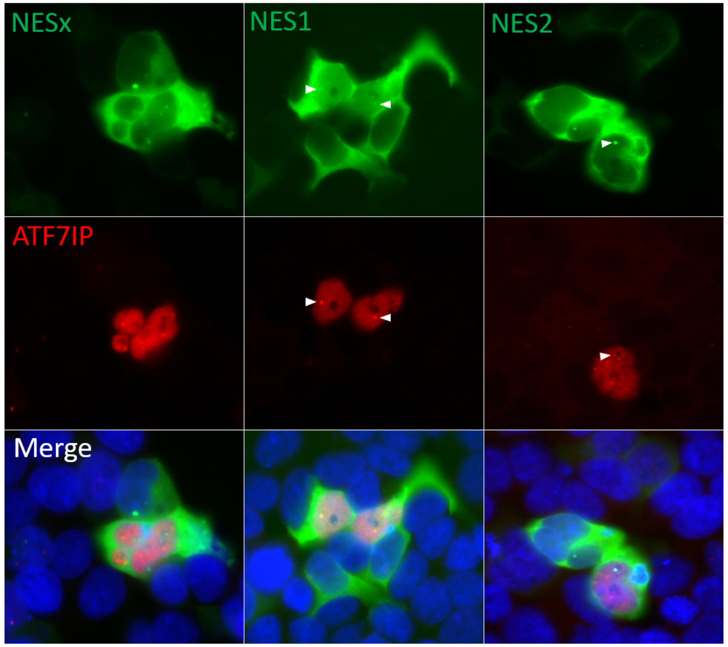

Figure S2. Effect of the NES motifs on SETDB1 movement in LMB-treated, ATF7IP-overexpressing 293T cells. Colocalization of ATF7IP-Flag and GFP signal is indicated by arrowheads.

Supplementary Figure S3

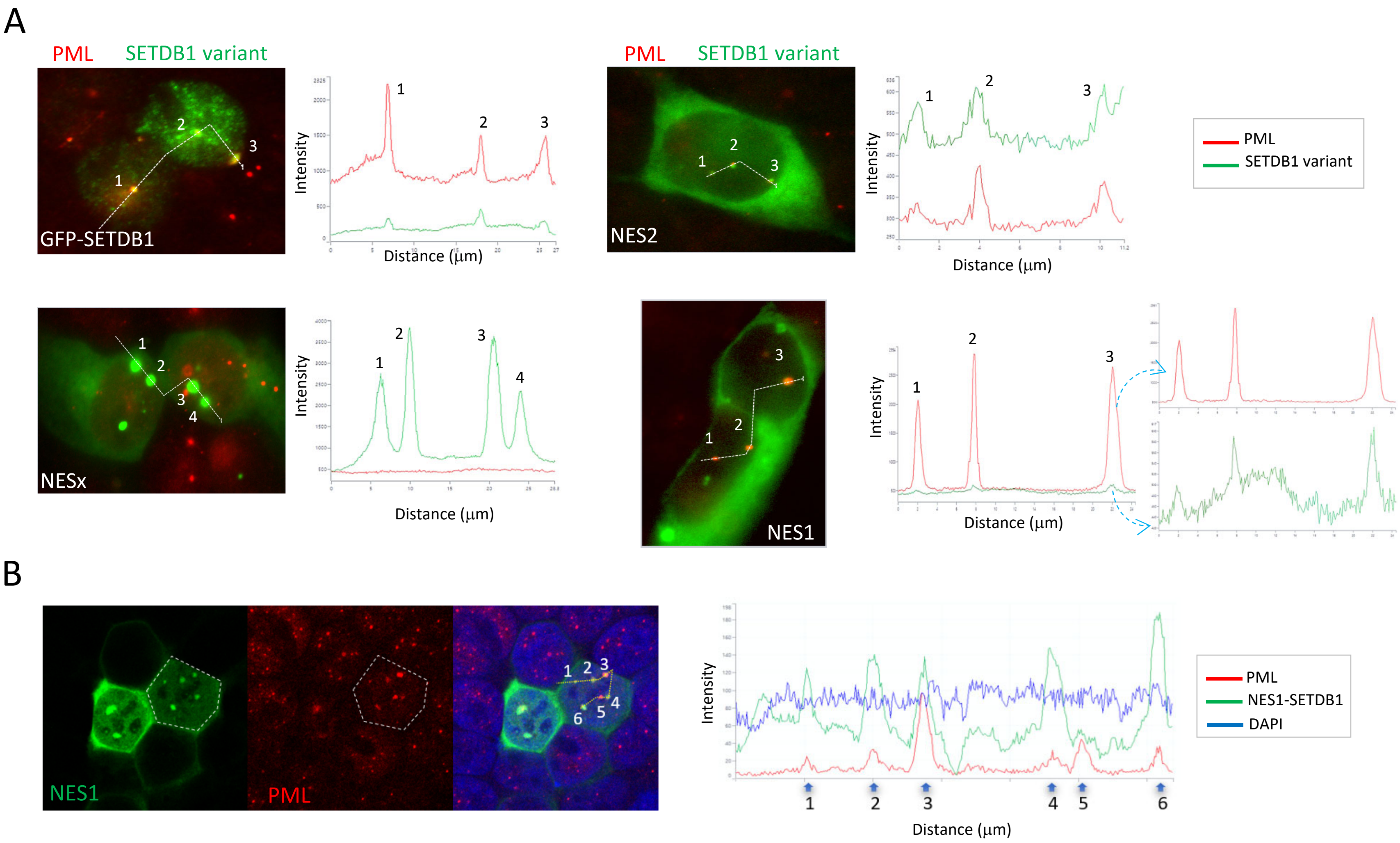

Figure S3. Localization of the dotted SETDB1 signal to the PML signal. Using the "Profile" tools in the Zen software (v3.4) from Carl Zeiss microscope, the fluorescence intensity of each individual dot in the images in **Fig. 3E** was quantified in different channels in **A**. The PML and GFP-SETDB1 profiles for the NES1 variant with a weak GFP signal were divided and individually analyzed. In **B**, a confocal picture of cells expressing NES1-SETDB1 also provided a signal intensity profile. The NES variant constructs and ATF7IP-Flag were co-transfected into 293T cells in **A** and **B**, and the cells were then stained with an anti-PML antibody. In accordance with the examination of the intensity profile, cells were numbered.

Supplementary Figure S4

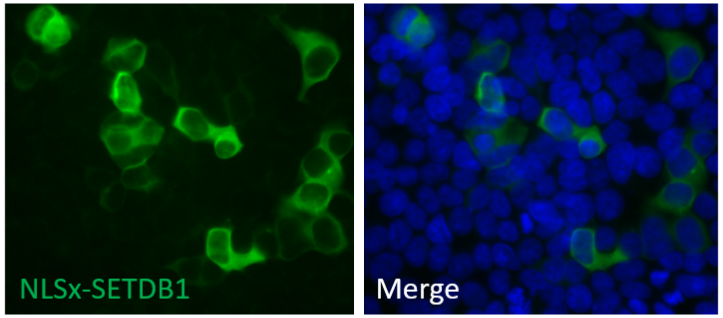

Figure S4. Cytoplasmic localization of NLSx-SETDB1 in 293T cells.
